# The -844 G>A PAI-1 Polymorphism Is Associated with Acute Coronary Syndrome in Mexican Population

**DOI:** 10.1155/2015/460974

**Published:** 2015-02-19

**Authors:** Ilian Janet García-González, Yeminia Valle, Elena Sandoval-Pinto, Emmanuel Valdés-Alvarado, Angélica Valdez-Haro, José Francisco Muñoz-Valle, Héctor Enrique Flores-Salinas, Luis Eduardo Figuera-Villanueva, Nory Omayra Dávalos-Rodríguez, Jorge Ramón Padilla-Gutiérrez

**Affiliations:** ^1^Instituto de Investigación en Ciencias Biomédicas, Centro Universitario de Ciencias de la Salud, Universidad de Guadalajara, 44350 Guadalajara, JAL, Mexico; ^2^Centro Universitario de Ciencias de la Salud, Universidad de Guadalajara, 44350 Guadalajara, JAL, Mexico; ^3^Centro Médico Nacional de Occidente, IMSS, 44350 Guadalajara, JAL, Mexico; ^4^Centro de Investigación Biomédica de Occidente, IMSS, 44350 Guadalajara, JAL, Mexico; ^5^Instituto de Investigación en Genética Humana, Centro Universitario de Ciencias de la Salud, Universidad de Guadalajara, 44350 Guadalajara, JAL, Mexico

## Abstract

*Background*. Acute coronary syndrome (ACS) has an important impact in public health with high morbidity and mortality. Prothrombotic and proinflammatory states are involved in the pathogenesis of the disease. Plasminogen activator inhibitor-1 (PAI-1) is the major inhibitor of the fibrinolysis and also is part of immune response. The -844 G>A gene polymorphism is related to increased PAI-1 protein levels. The aim of the study is to evaluate the association of -844 G>A PAI-1 polymorphism with ACS. *Methods*. A total of 646 individuals were recruited from Western Mexico: 350 unrelated healthy subjects and 296 patients with diagnosis of ACS. *Results*. The most important risk factor in our population was hypertension, followed by smoking. The genetic distribution showed an association of the A allele (OR = 1.27, *P* = 0.04) and AA genotype (OR = 1.86, *P* = 0.02) with ACS. The recessive model displayed similar results (OR = 1.76, *P* = 0.02). As additional finding, we observed significant differences in the genetic distribution of ACS dyslipidemic patients (OR = 1.99, *P* = 0.04). The A allele and AA genotype of -844 polymorphism of PAI-1 gene are risk factors for ACS. The AA genotype might be associated with the development of dyslipidemia in ACS patients.

## 1. Introduction

Acute coronary syndrome (ACS) is a growing concern around the world, a leading cause of death, and an important cause of disability [1, 2]. It is defined as a clinical group of entities which have as a common physiopathological mechanism the rupture of a plaque with thrombosis leading to acute myocardial ischemia. The clinical spectrum includes unstable angina, myocardial infarction with (STEMI) and without (NSTEMI) ST elevation [3].

ACS is a complex disease in which environmental and genetic factors play an interrelated role in its progress. It begins with an altered lipid metabolism and an inflammatory response, followed by the development of an atherosclerotic plaque. Nevertheless, in most cases the rupture of the plaque, formation of a thrombus, and the vessel lumen occlusion are required to trigger the acute myocardial event [4].

The haemostatic system is a key contributor of the thrombogenesis. It is widely known that multiple genes modulate the coagulation-fibrinolysis balance; therefore they could have an implication in the atherothrombotic vulnerability [5]. The biological function of PAI-1 as a fibrinolytic inhibitor situates the gene as an important candidate for ACS.

PAI-1 gene is part of the serpine superfamily. It is located in 7q21.3-q22 and contains 9 exons [6]. The encoded glycoprotein PAI-1 is the principal inhibitor of tissue (tPA) and urokinase (uPA) plasminogen activators, which are proteases responsible of fibrin degradation. Hence, PAI-1 inhibits the fibrinolysis and function as a thrombus stabilizer [6]. In addition, it has been observed that PAI-1 participates in the process of neovascular formation [7], also accepted as an important step in ACS physiopathology.

There is increasing evidence which indicates that higher levels of PAI-1 are an independent risk factor for the development of vascular disease, including cerebrovascular and cardiovascular disease [8, 9]. Moreover, several polymorphisms in PAI-1 gene has been identified and related to variation in plasmatic PAI-1 levels [10], including the -844 G>A polymorphism (rs2227631). The nucleotide substitution of guanine for adenine in the -844 position generates a consensus sequence for the Ets nuclear protein. Previous studies reported a relation of -844 G>A polymorphism with the change of gene expression and plasmatic protein levels [11, 12]. Therefore, current data sustains -844 G>A polymorphism as a plausible risk marker for ACS.

The aim of the present work is to analyze the association of the polymorphism -844 G>A with ACS in Western Mexican population.

## 2. Materials and Methods

### 2.1. Study Group and Population

The study consisted in a total of 646 individuals from Western Mexico, divided into two different groups: 296 ACS patients and 350 healthy subjects (HS). ACS patients were diagnosed in accordance with the criteria of the American College of Cardiology (ACC) [13]. The medical record of classical risk factors, defined according to the ACC, was registered and categorized as present or absent. The inclusion criteria of HS were absence of any chronic (type 2 diabetes mellitus (DM2), high blood pressure (HBP), cardiovascular diseases) or infectious diseases.

All the subjects were age-matched and recruited from the Hospital de Especialidades del Centro Médico Nacional de Occidente del Instituto Mexicano del Seguro Social (CMNO-IMSS). Only those individuals who for three generations, including their own, had been born in Western Mexico were considered.

### 2.2. Ethical Consideration

All subjects and patients agreed to participate and signed an informed written consent. The study was performed in accordance with the ethical guidelines of 2013 Declaration of Helsinki and with the approval of the ethics committee of the Centro Universitario de Ciencias de la Salud, UdeG (C.I. 069-2012).

### 2.3. DNA Analysis

Genomic DNA was extracted from peripheral blood according to salting out method [14]. The analysis of -844 G>A polymorphism was performed with the polymerase chain reaction (PCR). The primers sequences are forward, 5′-GTC TGG AGG AAG AGG ATA AAG GAC AA-3′, and reverse, 5′-CCT GAG GGC TCT CTT GTG TCA AC-3′. PCR amplification was carried out in a total volume of 25 *μ*L containing 1 *μ*g of gDNA, 1.25 U/*μ*L of Taq DNA polymerase (Invitrogen Life Technologies), supplied buffer enzyme 1x, 20 *μ*M of each oligonucleotide, 2.5 mM of MgCl_2_, and 2.5 mM deoxynucleotide triphosphate (dNTP Invitrogen Life Technologies).

The thermocycling conditions had an initial denaturation step of 3 min at 94°C, followed by 30 cycles of 20 s each at 94°C, 57°C, and 72°C, with a final extension step of 1 min at 72°C. PCR products of 510 base pairs (bp) were digested for 1 h at 37°C with 3 U of* Xho*I restriction enzyme (New England Biolabs, Beverly, MA). The G allele generates an* Xho*I restriction site (5-′C/TCGA**G**-3′) which results in the production of two fragments of 364 and 146 bp. The -844 G>A PAI-1 polymorphism was analyzed by silver stained polyacrylamide gels (29 : 1; acrylamide : bisacrylamide). For the quality control of genotyping, one random sample of each homozygote genotype was made by automatized sequencing method (ABI PRISM 377 Genetic Analyzer, Applied Biosystems, Foster City, CA, USA).

### 2.4. Statistical Analysis

SPSS statistical package version 20.0, Excel 2010, and Genetic Data Analysis [15] were used for the statistical analysis. Continuous variables were expressed as means ± standard deviation (SD). The Kolmogorov-Smirnov test and sample size were considered in order to categorize parametrical (*t*-test, ANOVA) and nonparametrical tests (Spearman correlation, Kruskal-Wallis test, and Mann-Whitney *U* test). Qualitative data and Hardy-Weinberg equilibrium were analyzed with *χ* ^2^ test or Fisher's exact test, when applicable. Allele frequencies were determined by counting method. Dominant and recessive allele models were tested with *χ* ^2^ test. The significance level was <0.05. The measure of association was odds ratio (OR). Bivariate and multivariate logistic regression analyses were accomplished to adjust the risk for every independent variable and to know the risk of polymorphism with ACS. Independent variables were categorized by risk factors, including diabetes, hypertension, smoking, dyslipidemia, and obesity.

The sample size was calculated based on the minor allele frequency of the -844 G>A PAI-1 polymorphism reported in Mexican Mestizo population by Padilla-Gutiérrez et al. [16] and we obtained at least 148 alleles using the Kelsey formula [17]. It means that, in order to detect differences, at least 74 individuals per group are needed with 95% confidence interval and statistical power of 80%.

## 3. Results

### 3.1. Description of Clinical Variables

HS and ACS patients groups had a mean age of 56.7 years (±8.3 SD) and 62.91 (±11.81 SD), respectively. As it is depicted in Table 1, the ACS group consisted of 3 times more males than females. The cardiac biomarkers values, including troponin T, creatine phosphokinase (CK), and CK-MB, were increased. Similarly, plasmatic glucose was above normal values.

The most prevalent risk factor was hypertension, followed by smoking and type 2 diabetes (DM2). Treatment included administration of antiplatelet (acetylsalicylic acid, clopidogrel), heparin, statins (atorvastatin), and antihypertensive drugs.

*Analysis of the -844 G>A Polymorphism*. The HS group was in Hardy-Weinberg equilibrium (*P* = 0.29). We compared the HS frequencies with other populations. The A allele prevalence was 23% in Sub-Saharan African, 27% in Mexican, 28% in Afro-American, 41% in Hispanic, 49% in Asian, and 56% in European populations [16, 18]. The genetic distribution of the -844 G>A polymorphism in this cohort was different to those reported in Sub-Saharan African, Asian, and Europeans populations (*P* < 0.01).

Allele and genotype frequencies were compared between HS and ACS groups (Table 2). The distributions were statistically different, in which the A allele (OR = 1.27, *P* = 0.04) and the AA genotype (OR = 1.86, *P* = 0.02) were more prevalent in ACS contrasted to HS group. We also included dominant and recessive models in the analysis. In the former model no association was found, whereas recessive model showed a significant association (OR = 1.76, *P* = 0.02).

Furthermore, ACS group was stratified by genotype and used to evaluate the most common risk factors and other characteristics (Table 3). We found a discrete association with two variables: dyslipidemia and smoking. The homozygous carriers for the A allele had more probability to have dyslipidemia than carriers of the G allele (OR = 1.99, *P* = 0.04) in the recessive model. The variables overweight/obesity, DM2, dyslipidemia, and HBP were analyzed by logistic regression with a recessive model as dependant variable, keeping their significance in ACS dyslipidemic patients with the AA genotype (*P* = 0.04). No differences were found among genetic frequencies in other characteristics.

## 4. Discussion

Atherothrombotic and neovascularization process are important mechanisms of cardiovascular disease in ACS. As highlighted above, PAI-1 molecule participates in both processes [7]. Relevant properties of PAI-1 in angiogenesis are related to its expression in endothelial cells and the regulation of their adhesion, migration, and growth [7]. Moreover, it restricts the excessive destruction of extracellular matrix in angiogenesis [7]. With regard to thrombotic process, PAI-1 inhibits tPA and uPA and hence diminishes fibrin degradation, maintaining a more stable thrombus and increasing the risk of vessel occlusion [19]. Consequently, PAI-1 is considered an important molecule in ACS.

The age and gender are the most important factors for ACS, in which an advanced age and male sex are considered with an increased risk [20]. In our population the mean age and the proportion of male/female are consistent with previous reports [20]. The increased cardiac biomarkers values and hyperglycemic states can be explained because most of our patients were recruited during the hospital admission in the acute event. It has been reported that hyperglycemia can be found in even more than 50% of hospitalized patients with ACS and it is recognizable predictor factor of mortality [21]. Cardiac biomarkers are part of the diagnosis of NSTEMI and STEMI in ACS [13], in consequence their increased levels were an expected finding.

The most common risk factor among our population was HBP, followed by smoking, DM2, and dyslipidemia. In opposition with our findings, RENASICA study displayed the prevalence of smoking above HBP [20]. An explanation for discrepancy between both results is the fact that our population belongs to a more delimitated region and the sample is smaller. In contrast, other studies had similarities in the occurrence of risk factors with our population [22].

As it was established previously, we found differences in the genetic distribution in the HS group compared with other populations, including Europeans, Asians, and Sub-Sahara Africans, but not with the African American group and a previous report of Western Mexicans [16]. The own genetic structure of the Mexican Mestizo population, composed mainly with Spanish (53.2%) and American native (30.8%) genes, can explain these differences [23]. The acquired data emphasizes the value of genetic variability recognition among populations.

In our study, we found an association of -844 G>A polymorphism with ACS. The A allele was 1.27 times more frequent in patients than HS (36.7 versus 31.3%, OR = 1.27, *P* = 0.04). The AA genotype carriers had 1.86 more susceptibility to ACS (14.2 versus 8.6%, OR = 1.86, *P* = 0.02). In accordance with the genetic models, two copies of the A allele are required to modify ACS susceptibility (OR = 1.76, *P* = 0.02).

PAI-1 is a remarkable molecule which modulates the development of atherosclerosis and cardiovascular disease [19, 24]. Interrelated mechanisms comprise an altered fibrinolysis toward a prothrombotic state, changes in vascular remodeling, and the association with other risk factors [19]. Although few studies have investigated the relationship of -844 G>A polymorphism with heart disease, Fu and collaborators in 2001 did not find a relation between this polymorphism and myocardial infarction [25]. Nevertheless, Abboud and collaborators observed an augmented risk of A allele carries with myocardial infarction [26], whereas others found it in coronary heart disease and myocardial infarction in nonsmokers [10, 27].

The base change of G for A in position -844 has been associated with different diseases, including venous thrombosis, preeclampsia, stroke, rheumatoid arthritis, systemic lupus erythematosus, metabolic syndrome, and myocardial infarction. The involvement in vascular, metabolic, inflammatory, and/or thrombotic systems is connection between these diseases and ACS [9, 12, 16, 26, 28–30].

Furthermore, previous reports supported the relationship existing between -844 G>A polymorphism and plasmatic PAI-1 levels, where A allele and AA genotype are associated with superior amounts of protein [10, 26, 31, 32] and mRNA [28]. As it is stated above, the variant generates an Ets-1 consensus binding sequence in PAI-1 promoter gene with an increase in the transcription rate [33]. The involvement of Ets-1 transcription factor in vascular inflammation and remodeling pathways [34] predisposes to plaque rupture and related complications. It is known that an increase in the plasma levels of PAI-1 is associated with coronary heart disease [24], which gives support to our findings with the genotype. Nonetheless others studies have not been found the association [35] or it was found in haplotype analysis with other loci [36, 37]. It is important to mention that we were not able to measure the plasmatic levels of PAI-1 or the mRNA.

In addition, the recessive model indicated a greater risk of ACS patients to have dyslipidemia in AA genotype carriers in comparison to AG and GG genotypes, which suggest the necessity of both alleles to modify dyslipidemia risk. In 2002, Haselbauer and collaborators did not find an association of the -844 G>A polymorphism with coronary artery disease or myocardial infarction; however the Gensini Score, which evaluates the collateral circulation of coronary arteries, was higher in carriers of AA genotype in patients with unfavorable lipid profile [38]. de la Cruz-Mosso and coworkers found an association of A allele and AA genotype with a dyslipidemic profile [9]. Moreover, they found a genetic association with obesity, which contrasts with our results and can be explained by the differences in selection criteria, specially age and diagnosis. Also in this concern a limitation in our work was the lack of rigorous scrutiny of our patients by body mass index that could mask the real relationship of obesity and the genotype.

## 5. Conclusions 

The AA genotype carries of the -844 G>A PAI polymorphism have 1.86 more susceptibility to develop SCA. Also, under the recessive genetic model this polymorphism was associated with dyslipidemia in SCA.

## Figures and Tables

**Table 1:** Demographic and clinical assessments in ACS patients and HS.

ACS parameter	Average	±SD

Ratio male/female	3.48	
Age (years)	62.91	±11.81
Glucose (mg/dL)	164.19	±69.47
CK (IU/L)	892.52	±1462.96
CK-MB (IU/L)	52.00	±163.45
Troponin T (ng/mL)	7.22	±14.10
Creatinine (mg/dL)	1.05	±0.96
Hemoglobin (mg/dL)	13.62	±2.17

HS parameters	Average	±SD

Ratio male/female	1.09	
Age (years)	56.76	±8.37
Glucose (mg/dL)	85.53	±12.92
Triglycerides (mg/dL)	102.50	±24.59
HDL (mg/dL)	49.99	±9.99
LDL (mg/dL)	90.30	±15.4

ACS treatment	*n*	(%)

ACE inhibitors	161	(54.39)
Acetylsalicylic acid	282	(95.27)
ARB	33	(11.15)
BB	151	(51.01)
CCB	36	(12.16)
Clopidogrel	221	(74.66)
Heparin	237	(80.07)
Isosorbide	39	(13.18)
Spironolactone	101	(34.12)
Statins	242	(81.76)

ACS Risk factor	*n*	(%)

Obesity	116	(39.19)
DM2	143	(48.31)
Dyslipidemia	126	(42.57)
HBP	179	(60.47)
Smoking	170	(57.43)
COPD	8	(2.70)

ACE: angiotensin converting enzyme; ACS: acute coronary syndrome; ARB: angiotensin II receptor blockers; BB: beta blockers; CCB: calcium channel blockers; CK: creatine phosphokinase; CK-MB: creatine phosphokinase MB; COPD: chronic obstructive pulmonary disease; DM2: type 2 diabetes mellitus; HBP: high blood pressure; HS: healthy subjects; SD: standard deviation.

**Table 2:** Allele and genotype frequencies of -844 G>A polymorphism in ACS and HS.

	HS *n* (%)	ACS *n* (%)	OR (CI)	*P*
Allele				
G	481 (68.7)	375 (63.3)	—	—
A	219 (31.3)	217 (36.7)	1.27 (1.01–1.60)	0.04^*^
Genotype				
GG	161 (46.0)	121 (40.9)	—	—
GA	159 (45.4)	133 (44.9)	1.11 (0.80–1.55)	0.52
AA	30 (8.6)	42 (14.2)	1.86 (1.10–3.15)	0.02^*^
Dominant model				
GG	161 (46.0)	121 (40.9)	—	—
GA + AA	189 (54.0)	175 (59.1)	1.23 (0.90–1.69)	0.19
Recessive model				
GG + GA	320 (91.4)	254 (85.8)	—	—
AA	30 (8.6)	42 (14.2)	1.76 (1.07–2.89)	0.02^*^

ACS: acute coronary syndrome; CI: confidence interval; HS: healthy subjects; OR: odds ratio. ^*^Significant value.

**Table 3:** Clinical and demographic characteristics in the ACS patients related to -844 G>A polymorphism of PAI-1 gene by genetic models.

	ACS (*n* = 296)	Genotype							Dominant				Recessive			
		GG^a^ (*n* = 121)	GA (*n* = 133)	OR (CI)	*P*	AA (*n* = 42)	OR (CI)	*P*	GG^a^ (*n* = 121)	GA + AA (*n* = 175)	OR (CI)	*P*	GG + GA^a^ (*n* = 254)	AA (42)	OR (CI)	*P*
F/M	66/230	30/91	24/109	—	—	12/30	—	—	—	—	—	—	—	—	—	—
UA^b^	31	14	14	—	—	3	—	—	14	17	—	—	28	3	—	—
NSTEMI	40	12	18	1.06 (0.48–2.34)	0.88	10	1.43 (0.38–5.30)	0.59	12	28	1.13 (0.53–2.39)	0.76	30	10	1.38 (0.39–4.83)	0.61
STEMI	225	95	101	1.50 (0.53–4.25)	0.44	29	3.89 (0.87–17.48)	0.07	95	130	1.92 (0.72–5.11)	0.19	196	29	3.11 (0.78–12.48)	0.09
OB^c^	116	47	49	0.92 (0.55–1.53)	0.74	20	1.43 (0.71–2.90)	0.32	47	69	1.03 (0.64–1.65)	0.92	96	20	1.49 (0.78–2.89)	0.23
DM2^c^	143	65	60	0.71 (0.43–1.16)	0.17	18	0.65 (0.32–1.31)	0.23	65	78	0.69 (0.44–1.10)	0.12	125	18	0.77 (0.40–1.49)	0.45
HBP^c^	179	77	76	0.76 (0.46–1.26)	0.29	26	0.93 (0.45–1.92)	0.84	77	102	0.79 (0.49–1.29)	0.35	153	26	1.07 (0.55–2.10)	0.84
DYS^c^	126	52	50	0.79 (0.48–1.32)	0.38	24	1.77 (0.87–3.59)	0.11	52	74	0.97 (0.61–1.55)	0.91	102	24	1.99 (1.03–3.85)	0.04^*^

ACS: acute coronary syndrome; DM2: type 2 diabetes mellitus; DYS: dyslipidemia; F: female; HBP: high blood pressure; M: male; NSTEMI: non-ST elevation myocardial infarction; OB: obesity/overweight; SMO: smoking; STEMI: ST elevation myocardial infarction; UA: unstable angina. ^a^Reference genotype. ^b^UA used as reference with ACS types.^ c^Not present as reference variable. ^*^Significant value.

## Abbreviations

ACC: American College of Cardiology
ACS: Acute coronary syndrome
bp: Base pair
CMNO-IMSS: Centro Médico Nacional de Occidente del Instituto Mexicano del Seguro Social
DM2: Type 2 diabetes mellitus
CK: Creatine phosphokinase
HBP: High blood pressure
HS: Healthy subjects
NSTEMI: Myocardial infarction without ST elevation
OR: Odds ratio
PAI-1: Plasminogen activator inhibitor 1
PCR: Polymerase chain reaction
STEMI: Myocardial infarction with ST elevation
tPA: Tissue plasminogen activator
uPA: Urokinase plasminogen activator.
